# Molecular Genetics and Cytogenetics in Cancer

**DOI:** 10.4061/2011/835053

**Published:** 2012-02-26

**Authors:** José María Sayagués, Sergio Roa, Norma C. Gutierrez, Ilana Zalcberg Renault

**Affiliations:** ^1^Centro de Investigación del Cáncer, (IBMCC-CSIC/USAL) and Instituto de Investigación Biomédica de Salamanca (IBSAL), Universidad de Salamanca, 37007 Salamanca, Spain; ^2^Department of Cell Biology, Albert Einstein College of Medicine, 1300 Morris Park Avenue, Bronx, NY 10461, USA; ^3^Hospital Universitario de Salamanca, Paseo de San Vicente 58-182, 37007 Salamanca, Spain; ^4^Laboratory of Molecular Biology, Bone Marrow Transplantation Center (CEMO), National Cancer Institute, 20230-130 Rio de Janeiro, RJ, Brazil

This special issue of Genetics Research International focuses on *molecular genetics and cytogenetics in cancer*. The hunt for informative disease-related genetic biomarkers may become so valuable for the differential diagnosis, prognosis, and disease monitoring of cancer patients that numerous researchers are committed to this challenge. Indeed, a colossal effort of current molecular medicine to progress in the understanding of the hallmarks of cancer is focusing on the field of biomarker discovery through genetic and cytogenetic profiling. Ambitious initiatives such as the Cancer Genome Atlas (http://cancergenome.nih.gov/) or the Cancer Genome Anatomy Project (http://cgap.nci.nih.gov/cgap.html) from the US National Institutes of Health (NIH) are devoted to provide collaborative platforms to better comprehend and fight the malignant signature of cancer. 

The fast development of high-throughput technologies is bringing to biomedical research more affordable and high-resolution ways to explore whole genomes, chromosomes, genes, proteins, RNA, and metabolites. In consequence, numerous genomic datasets accumulate in international repositories, such as GEO from the US National Center for Biotechnology Information (NCBI), and around 500,000 clonal chromosomal abnormalities belonging to more than 60,000 human neoplasms are described in the latest catalogue of chromosome aberrations in cancer. However, translational application of biomarker discovery still has to face its ultimate challenge: incorporation into routine clinical practice. 

We are under the pressure to combine the multidisciplinary efforts of basic, clinical, and computational research fields to identify meaningful genetic profiles that can be used as powerful tools for diagnosis, prognosis, and treatment. Every patient who suffers from cancer hopes that our research will deliver alternatives. For scientists, the identification of the specific genetic abnormalities responsible for tumor diversity and heterogeneity is crucial to shed light on the molecular mechanisms of cancer and the identification of druggable targets and functional pathways. For clinicians, the appropriate molecular categorization of patients would allow to enroll them in tailored—more adequate and less toxic—treatments, improving healthcare and optimizing medical costs.

Ultimately, any effort to (i) frame the important unsolved problems, (ii) to review what is known about the molecular pathways involved in the pathogenesis of distinct types of cancer, (iii) to catalog recurrent genetic alterations that could serve as biomarkers, (iv) to suggest standardization of routine protocols of diagnosis and molecular detection, and (v) to rank available or potential drugs and their targets, is decisive to help molecular medicine in its challenge against cancer. This is the scope of this special issue where the biographies of some of the most aggressive hematological and solid tumors are discussed. The journal Genetics Research International is serving here as a forum for discussion and review of major genetic risks involved in breast cancer metastasis to the brain as well as in the evolution of myxoid soft-tissue sarcomas and parathyroid tumors. The importance of applying genetic protocols in the risk-based stratification of multiple myeloma is also reviewed here, and new evidences supporting genetic aberrations in a rare variant of T-cell lymphomas known as Sézary syndrome are presented. Finally, myeloid malignancies are revealed as a paradigm to discuss the role of dicentric chromosomes as instigators of genomic instability and suggest a model for their implication in the formation of unbalanced translocations in malignancy.

Breaking the barriers of cancer is a difficult task that is continuously challenging the research community.

We, the editors of this special issue, are hopeful that the ideas presented here can inspire the critical thinking of the specialized readers and contribute to the common efforts to better understand and fight the multiple faces of cancer.

**José María Sayagués** **José María Sayagués** **Sergio Roa** **Sergio Roa** **Norma C. gutierrez** **Norma C. gutierrez** **Ilana Zalcberg Renault** **Ilana Zalcberg Renault**

